# Comparison of the predictive value of CONUT, NLR, and PNI for 6-month and 1-year mortality in middle-aged and older adults with hip fractures: a retrospective study

**DOI:** 10.3389/fnut.2026.1780521

**Published:** 2026-05-28

**Authors:** Zhi-qin Chen, Liang Lin, Chang-zhi Huang, Yong-qiang Zheng, Ming-ming Lin, Yang-zhen Fang, Ze-feng Wang, Can-hong Zhang, Jin-shan Zhang, Xiao-feng Liu

**Affiliations:** 1 Department of Orthopedic, Jinjiang Municipal Hospital (Shanghai Sixth People's Hospital Fujian), Quanzhou, Fujian, China; 2 Jinjiang Municipal Hospital, Teaching Hospital of Huaqiao University, Jinjiang, Fujian, China; 3 Department of Orthopedic, The First Hospital of Putian City, Putian, Fujian, China; 4 Department of Orthopedic Surgery, The First Affiliated Hospital of Fujian Medical University, Fuzhou, China; 5 Ningde Clinical Medical College of Fujian Medical University, Ningde, Fujian, China; 6 Department of Joint Surgery and Sports Medicine, Ningde Municipal Hospital of Ningde Normal University, Ningde, Fujian, China; 7 Department of Orthopedic Surgery, Quanzhou First Hospital Affiliated to Fujian Medical University, Quanzhou, Fujian, China

**Keywords:** CONUT score, hip fracture, middle-aged and older adults, NLR, nutritional status, PNI, short-term mortality

## Abstract

**Objective:**

This study aimed to compare the predictive value of three preoperative nutritional status scores—Controlling Nutritional Status (CONUT) score, Neutrophil-to-Lymphocyte Ratio (NLR), and Prognostic Nutritional Index (PNI)—for 6-month and 12-month mortality in middle-aged and older adults with hip fractures.

**Methods:**

A retrospective cohort study was conducted on 198 middle-aged and older adults (≥50 years) who underwent surgical treatment for hip fractures at Jinjiang Municipal Hospital (Shanghai Sixth People’s Hospital Fujian) between January 2020 and December 2021. Preoperative laboratory data were used to calculate CONUT, NLR, and PNI scores. Receiver operating characteristic (ROC) curves were used to evaluate predictive efficacy, and Kaplan–Meier analysis was used to compare mortality across nutritional status subgroups.

**Results:**

Thirty patients (15.15%) died within 6 months and 40 (20.20%) within 12 months. Multivariate analysis suggested high CONUT score was independently associated with short-term mortality. The CONUT score significantly predicted 6-month (AUC = 0.620, 95% CI: 0.531–0.710, *p* = 0.036) and 12-month mortality (AUC = 0.617, 95% CI: 0.531–0.702, *p* = 0.022), with an optimal cut-off of 2.5. Patients with CONUT ≥3 had higher 6-month (20.16% vs. 5.80%) and 12-month mortality (25.58% vs. 10.14%) than those with CONUT <3.

**Conclusion:**

Among the three scores, the CONUT score was associated with better predictive performance for 6-month and 12-month mortality in middle-aged and older adults with hip fractures. Preoperative CONUT score assessment may help identify high-risk patients and support targeted nutritional interventions. NLR and PNI showed limited value in predicting short-term mortality in this population.

**Trial registration:**

This study has been registered in national medical research registration and filing information system of China, www.medicalresearch.org.cn, Trail registration number: MR-35-24-034127. Registration date: 28 August 2024.

## Introduction

Hip fracture is a prevalent, severe musculoskeletal injury among middle-aged and older adults, with rapidly increasing incidence worldwide due to population aging (1, 2). It is characterized by high rates of disability, complications, and mortality, placing a heavy burden on patients, families, and healthcare systems (3). Short-term mortality, especially at 6 and 12 months after surgery, remains a critical indicator of postoperative outcomes and a major clinical concern.

Nutritional status is a modifiable factor closely related to postoperative recovery and survival. Malnutrition is common in elderly patients with hip fracture and is linked to impaired immune function, delayed healing, and higher risks of complications and death (4).

Several scoring systems, including the Controlling Nutritional Status (CONUT) score (5), Neutrophil-to-Lymphocyte Ratio (NLR) (6), and Prognostic Nutritional Index (PNI) (7), have been used to evaluate nutritional and inflammatory status and show potential prognostic value in surgical patients. However, direct comparisons of these three scores for predicting short-term mortality in middle-aged and older adults with hip fracture remain limited (8, 9).

The aim of this study was to compare the predictive value of preoperative CONUT, NLR, and PNI for 6-month and 12-month mortality in middle-aged and older adults undergoing hip fracture surgery.

## Materials and methods

### Study population

**Figure 1:**
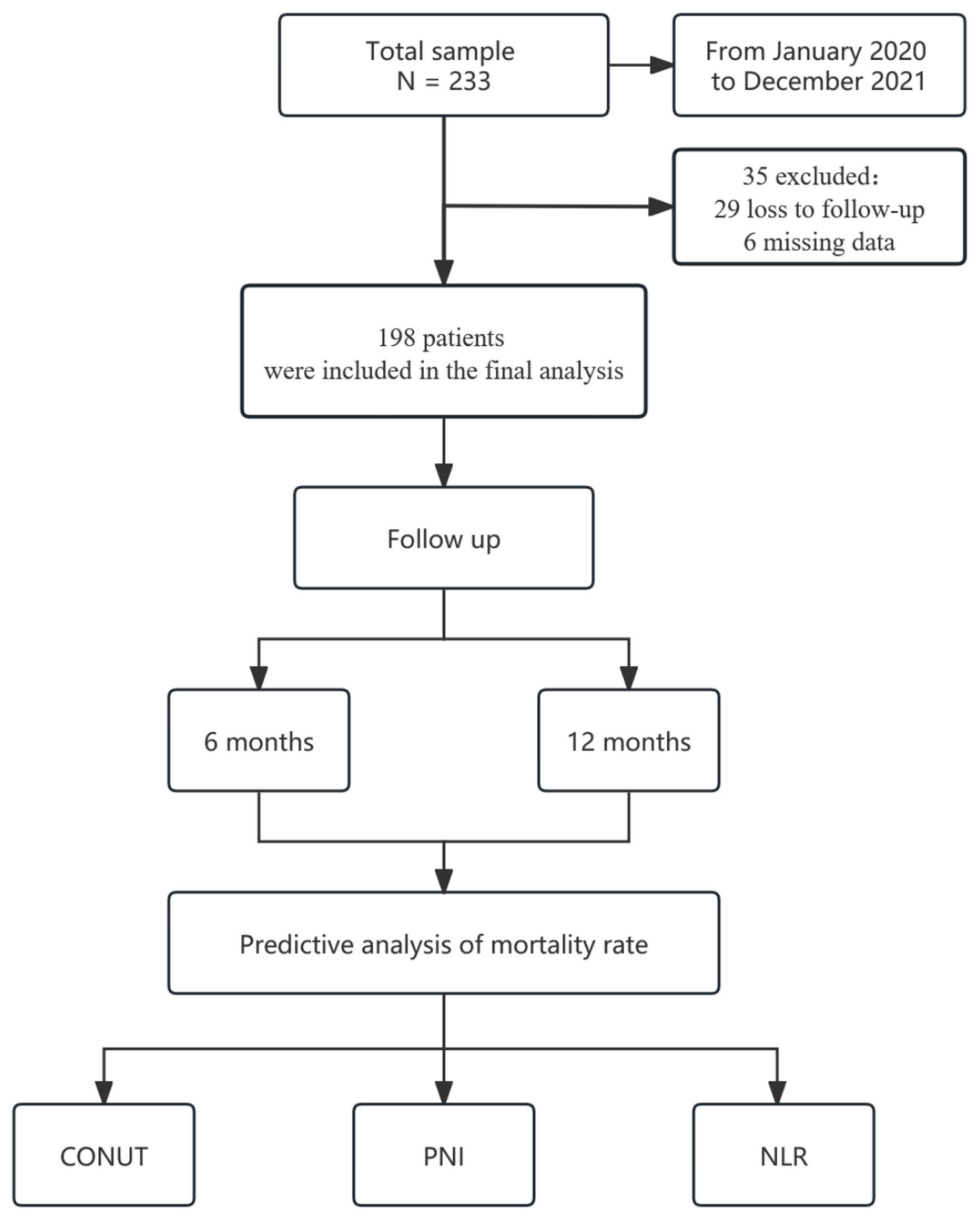
Study cohort flow diagram.

This retrospective cohort study enrolled middle-aged and older adults (≥50 years) with hip fractures who underwent surgical treatment at Jinjiang Municipal Hospital (Shanghai Sixth People’s Hospital Fujian) between January 2020 and December 2021. The inclusion criteria were as follows: (1) diagnosis of hip fracture (femoral neck or intertrochanteric fracture) confirmed by X-ray or CT imaging; (2) age ≥50 years; (3) availability of complete preoperative laboratory data for calculating CONUT, NLR, and PNI scores; (4) completion of 6-month and 12-month postoperative follow-up. Exclusion criteria included: (1) pathological fractures caused by tumors or infections; (2) multiple fractures or high-energy trauma (e.g., motor vehicle accidents); (3) incomplete clinical or follow-up data; (4) preoperative receipt of nutritional support or anti-inflammatory therapy that might affect laboratory indicators; (5) terminal diseases with an expected survival <6 months. The study was approved by the Ethics Committee of Jinjiang Municipal Hospital (Shanghai Sixth People’s Hospital Fujian) (Approval No.: jjsyyyxll-2023031), and informed consent was obtained from all patients or their legal representatives. The flow diagram of the study cohort is presented in Figure 1, which clearly illustrates the patient selection process.

### Data collection

Clinical data were extracted from the hospital’s electronic medical record system, including demographic information (age, gender), fracture-related details (fracture type, surgical method, time from injury to surgery), comorbidities (hypertension, diabetes mellitus, cardiovascular disease, chronic respiratory disease, etc.), and preoperative laboratory results (serum albumin, total lymphocyte count, total cholesterol, neutrophil count, hemoglobin). Follow-up was conducted via outpatient visits or telephone interviews at 6 and 12 months postoperatively to record survival status. The primary outcomes were 6-month and 12-month all-cause mortality.

### Calculation of nutritional status scores

CONUT score (Table 1): Calculated based on three parameters (10): serum albumin (g/dL) ≥ 3.5 (0 points), 3.0–3.4 (2 points), 2.5–2.9 (4 points), <2.5 (6 points); total lymphocyte count (/μL) ≥ 1,600 (0 points), 1,200–1,599 (1 point), 800–1,199 (2 points), <800 (3 points); total cholesterol (mg/dL) ≥ 180 (0 points), 140–179 (1 point), 100–139 (2 points), <100 (3 points). The total score ranges from 0 to 12, with higher scores indicating poorer nutritional status.

**Table 1:** Assessment of the nutritional status using CONUT.

Parameter	Malnutrition status			
	Normal	Light	Moderate	Severe
Serum albumin (mg/dL)	≧3.5	3.0–3.49	2.5–2.99	<2.5
Albumin score	0	2	4	6
Total cholesterol level (mg/dL)	≧180	140–179	100–139	<100
Cholesterol score	0	1	2	3
Total lymphocyte count (/mm^3^)	≧1,600	1,200–1,599	800–1,199	<800
Lymphocyte score	0	1	2	3
Total score	0–1	2–4	5–8	9–12

NLR: Calculated as the ratio of preoperative neutrophil count to lymphocyte count (6).

PNI: Calculated using the formula: PNI = 10 × serum albumin (g/dL) + 0.005 × total lymphocyte count (/mm^3^) (11).

### Bias minimization

To minimize selection bias and information bias, strict inclusion and exclusion criteria were applied consistently to all eligible patients. All clinical data and laboratory results were independently extracted by two trained researchers who were blinded to patient outcomes. Any discrepancies were resolved by a third senior investigator. Survival status at 6 and 12 months was assessed via standardized outpatient or telephone follow-up, with records independently verified to ensure accuracy.

### Statistical analysis

Statistical analyses were performed using IBM SPSS Statistics 26.0 (SPSS Inc., Chicago, IL, USA). The normality of continuous variables was examined using the Shapiro–Wilk test, combined with visual inspection of histograms, skewness, and kurtosis. Normally distributed data were presented as mean ± standard deviation (SD) and compared using independent-samples *t*-tests. Non-normally distributed data were presented as median (interquartile range, IQR) and compared using the Mann–Whitney *U* test. Categorical variables were presented as counts (percentages) and compared using the chi-square test.

Receiver operating characteristic (ROC) curves were constructed to evaluate the predictive value of CONUT, NLR, and PNI for 6-month and 12-month mortality. The optimal cutoff value was determined using the Youden index. Kaplan–Meier curves and log-rank tests were used to compare survival differences between groups. Multivariate Cox regression analysis was performed to identify independent risk factors for mortality.

All tests were two-sided, and exact *p*-values are reported throughout the manuscript. A *p*-value <0.05 was considered statistically significant, and *p* < 0.001 was used only for results with extremely high statistical significance. All statistical procedures were verified and reviewed by a professional biostatistician.

## Results

### Baseline demographics and characteristics of patients by survival at 6 months

A total of 198 eligible patients were included in the final analysis, with 30 patients (15.15%) dying within 6 months postoperatively and 168 patients (84.85%) surviving. As shown in Table 2, the baseline characteristics differed significantly between the two groups in terms of age, gender, comorbidity status, and hypertension prevalence (all *p* < 0.05).

**Table 2:** Baseline demographics and characteristics of patients by survival at 6 months.

Parameter	Died at 6 months (*N* = 30)	Survived at 6 months (*N* = 168)	*t*/*χ*^2^ value	*p* value
Age	81.73 ± 8.00	77.08 ± 12.61	2.654	0.010
Male/female	16/14	55/113	4.694	0.030
Duration of surgery, median (Q1–Q3)	72 (55–95)	75 (57–101)	0.139	0.890
Hospitalization length, median (Q1–Q3)	11 (6–13)	12 (7–13)	0.208	0.836
Comorbidities	30 (100.00%)	138 (82.14%)	6.314	0.012
DM2	7 (23.33%)	26 (15.48%)	1.131	0.288
Hypertension	22 (73.33%)	75 (44.64%)	8.385	0.004
Pneumonia	6 (20.00%)	43 (25.60%)	0.428	0.513
SBP (mmHg)	153.47 ± 28.02	151.53 ± 25.40	0.378	0.706
DBP (mmHg)	77.83 ± 11.17	77.98 ± 11.91	0.064	0.949
WBC (10^9^/L)	8.98 ± 2.68	9.65 ± 2.56	1.296	0.196
Hemoglobin (g/L)	110.27 ± 24.78	116.94 ± 21.77	1.514	0.132
Albumin (g/L)	37.28 ± 4.50	37.65 ± 5.57	0.347	0.729
ALP (u/L)	107.20 ± 73.14	88.93 ± 58.42	1.514	0.132
ALT (g/L)	19.79 ± 16.59	19.20 ± 15.21	0.193	0.848
Uric acid (μmol/L)	305.17 ± 128.78	314.16 ± 107.65	0.408	0.684
Blood calcium (mmol/L)	2.21 ± 0.16	2.25 ± 0.16	1.258	0.210
Serum phosphorus (mmol/L)	1.01 ± 0.18	1.01 ± 0.20	0.029	0.977
TC (mmol/L)	4.24 ± 1.19	4.43 ± 1.08	0.879	0.381
TG (mmol/L)	1.01 ± 0.36	1.03 ± 0.49	0.253	0.800
HDL (mmol/L)	1.30 ± 0.40	1.34 ± 0.40	0.549	0.584
LDL (mmol/L)	2.60 ± 1.02	2.75 ± 0.90	0.811	0.419

Data are presented as mean ± SD or as number (%). RDW, red blood cell distribution width. DM2, type 2 diabetic mellitus. SBP, systolic blood pressure. DBP, diastolic blood pressure. WBC, white blood cell. ALP, alkaline phosphatase. ALT, alanine aminotransferase. TC, total cholesterol. TG, triglyceride. HDL, high-density lipoprotein. LDL, low-density lipoprotein.

The non-survivor group had a significantly higher mean age than the survivor group (81.73 ± 8.00 years vs. 77.08 ± 12.61 years, *p* = 0.010). In terms of gender distribution, the proportion of males was higher in the non-survivor group (53.33% vs. 32.74%, *p* = 0.030). All patients in the non-survivor group had at least one comorbidity, which was significantly higher than the survivor group (100.00% vs. 82.14%, *p* = 0.012). Specifically, the prevalence of hypertension in the non-survivor group was markedly higher (73.33% vs. 44.64%, *p* = 0.004).

However, there were no statistically significant differences between the two groups in terms of surgical duration (87.36 ± 63.54 min vs. 86.01 ± 41.22 min, *p* = 0.890), length of hospitalization (11.87 ± 7.01 days vs. 12.14 ± 6.49 days, *p* = 0.836), and other comorbidities such as type 2 diabetes mellitus (DM2) and pneumonia (both *p* > 0.05). Additionally, no significant differences were observed in preoperative laboratory indicators including white blood cell count (WBC), hemoglobin, albumin, alkaline phosphatase (ALP), alanine transferase (ALT), blood calcium, serum phosphorus, and lipid profiles [total cholesterol (TC), triglycerides (TG), high-density lipoprotein (HDL), low-density lipoprotein (LDL)] (all *p* > 0.05).

### Comparison of the predictive efficacy of different evaluation indicators for short-term mortality in hip fracture

To explore the predictive value of nutritional status scores for short-term postoperative mortality, we compared the distribution of CONUT, PNI, and NLR between survivors and non-survivors at 6 and 12 months postoperatively, with results presented in Tables 3, 4.

**Table 3:** Comparison of the predictive efficacy of different evaluation indicators for 6-month mortality in hip fracture.

Parameter	Non-survivors (*N* = 30)	Survivors (*N* = 168)	*p*-value
CONUT	3.83 ± 1.29	3.26 ± 2.02	0.047
Serum albumin level (mg/dL)	3.73 ± 0.45	3.76 ± 0.57	0.729
Total cholesterol level (mg/dL)	163.90 ± 46.05	171.26 ± 41.58	0.381
Total lymphocyte count (/mm^3^)	1138.67 ± 362.17	1103.69 ± 463.80	0.696
PNI	42.97 ± 4.84	43.17 ± 5.86	0.862
Serum albumin level (g/dL)	37.28 ± 4.50	37.65 ± 5.57	0.729
Total lymphocyte count (10^9/L)	1.14 ± 0.36	1.10 ± 0.46	0.035
NLR	6.82 ± 3.77	8.86 ± 5.82	0.066
Neutrophil count (10^9/L)	7.00 ± 3.02	7.93 ± 2.34	0.058
Total lymphocyte count (10^9/L)	1.14 ± 0.36	1.10 ± 0.46	0.035

**Table 4:** Comparison of the predictive efficacy of different evaluation indicators for 12-month mortality in hip fracture.

Parameter	Non-survivors (*N* = 40)	Survivors (*N* = 158)	*p*-value
CONUT	3.78 ± 1.35	3.24 ± 2.04	0.049
Serum albumin level (mg/dL)	3.72 ± 0.42	3.77 ± 0.57	0.608
Total cholesterol level (mg/dL)	159.40 ± 43.02	172.86 ± 41.75	0.072
Total lymphocyte count (/mm^3^)	1140.25 ± 369.92	1101.08 ± 467.90	0.623
PNI	42.90 ± 4.68	43.20 ± 5.95	0.770
Serum albumin level (g/dL)	37.20 ± 4.17	37.69 ± 5.69	0.608
Total lymphocyte count (10^9/L)	1.14 ± 0.37	1.10 ± 0.47	0.623
NLR	6.99 ± 3.55	8.94 ± 5.95	0.048
Neutrophil count (10^9/L)	7.18 ± 2.75	7.94 ± 2.38	0.085
Total lymphocyte count (10^9/L)	1.14 ± 0.37	1.10 ± 0.47	0.623

For 6-month mortality (Table 3), the non-survivor group had a significantly higher mean CONUT score than the survivor group (3.83 ± 1.29 vs. 3.26 ± 2.02, *p* = 0.047), indicating poorer nutritional status in patients who died within 6 months. Among the components of CONUT, serum albumin level, total cholesterol level, and total lymphocyte count showed no significant differences between the two groups (all *p* > 0.05). Regarding PNI, no statistically significant difference was observed between non-survivors and survivors (42.97 ± 4.84 vs. 43.17 ± 5.86, *p* = 0.862), despite the non-survivor group having a slightly lower PNI. For NLR, the non-survivor group tended to have a lower ratio than the survivor group (6.82 ± 3.77 vs. 8.86 ± 5.82), but the difference was marginally insignificant (*p* = 0.066). Notably, the total lymphocyte count (10^9^/L) was significantly higher in the non-survivor group (1.14 ± 0.36 vs. 1.10 ± 0.46, *p* = 0.035), while the neutrophil count showed no significant difference (*p* = 0.058).

For 12-month mortality (Table 4), consistent with the 6-month results, the non-survivor group had a significantly higher CONUT score than the survivor group (3.78 ± 1.35 vs. 3.24 ± 2.04, *p* = 0.049). Similar to the 6-month analysis, the individual components of CONUT (serum albumin, total cholesterol, total lymphocyte count) did not differ significantly between the two groups (all *p* > 0.05). PNI still showed no significant difference between non-survivors and survivors (42.90 ± 4.68 vs. 43.20 ± 5.95, *p* = 0.770). In contrast, NLR became significantly different between the two groups at 12 months: the non-survivor group had a lower NLR than the survivor group (6.99 ± 3.55 vs. 8.94 ± 5.95, *p* = 0.048). The total lymphocyte count (10^9^/L) remained comparable between the two groups (*p* = 0.623), and the neutrophil count showed no significant difference (*p* = 0.085).

Overall, CONUT score was significantly associated with both 6-month and 12-month postoperative mortality, while NLR only showed a significant association with 12-month mortality, and PNI had no significant predictive association with short-term mortality in this study population.

In the univariate analysis, age, male sex, hypertension, and high CONUT score were associated with increased 12-month mortality. To determine independent prognostic factors, multivariate Cox regression analysis was performed including these variables. As shown in Table 5, CONUT score was independently associated with an increased risk of 12-month mortality.

**Table 5:** Multivariate cox regression analysis for 12-month mortality.

Variable	HR	95% CI	*p*-value
Age	1.05	1.03–1.09	0.004
Sex	2.81	1.46–5.43	0.002
Hypertension	1.42	1.22–1.82	0.032
CONUT	1.53	1.13–2.16	0.045
PNI	1.02	0.97–1.07	0.530
NLR	0.95	0.90–1.02	0.153

### The ROC curve for different evaluation indicators in predicting short-term mortality

**Figure 2:**
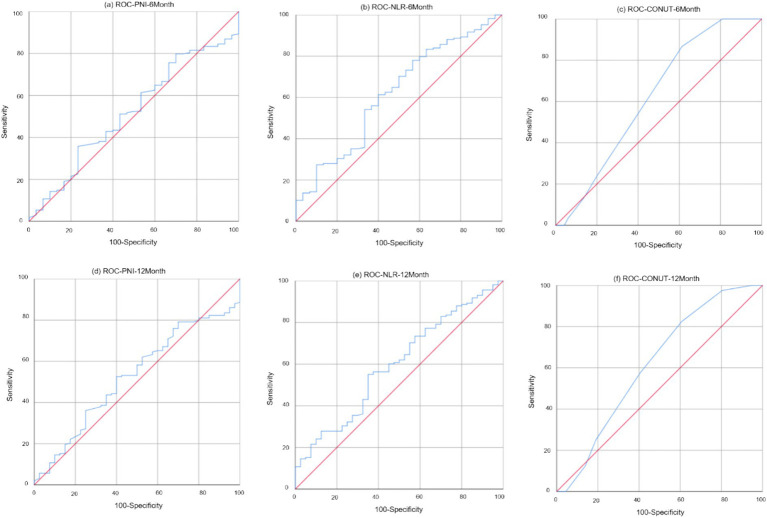
The ROC curves for different evaluation indicators in predicting mortality.

Receiver operating characteristic (ROC) curve analysis was performed to evaluate the predictive performance of CONUT, NLR, and PNI for 6-month and 12-month all-cause mortality. The optimal cutoff values, area under the curve (AUC), 95% CI, sensitivity, and specificity are summarized in Table 6 and illustrated in Figure 2.

**Table 6:** ROC Analysis Results for Significant Predictors.

Predictor	Month	Cut-off value	AUC	Sensitivity	Specificity	95% CI	*p*-value
PNI	6	44.9	0.520	35.7%	76.7%	0.412–0.628	0.055
	12	42.825	0.527	52.5%	60.0%	0.431–0.623	0.593
NLR	6	4.99	0.617	78.0%	43.3%	0.507–0.727	0.041
	12	7.039	0.597	55.1%	65.0%	0.501–0.693	0.058
CONUT	6	2.5	0.620	86.7%	38.7%	0.531–0.710	0.036
	12	2.5	0.617	82.5%	39.2%	0.531–0.702	0.044

For 6-month mortality, the CONUT score showed statistically significant predictive ability (AUC = 0.620, 95% CI: 0.531–0.710, *p* = 0.036). The optimal cutoff value determined by the Youden index was 2.5, with a sensitivity of 86.7% and specificity of 38.7%. NLR also achieved statistically significant prediction for 6-month mortality (AUC = 0.598, 95% CI: 0.502–0.694, *p* = 0.041). In contrast, PNI did not show a statistically significant predictive ability for 6-month mortality (*p* > 0.05).

For 12-month mortality, the CONUT score remained a significant predictor (AUC = 0.617, 95% CI: 0.531–0.702, *p* = 0.022), with the same optimal cutoff value of 2.5, yielding a sensitivity of 82.5% and specificity of 39.2%. NLR and PNI did not achieve statistically significant or clinically reliable predictive value for 12-month mortality.

Overall, the CONUT score exhibited the highest and most stable predictive performance for both 6-month and 12-month mortality among the three scores. These results support the use of preoperative CONUT score as a practical and effective tool for risk stratification in middle-aged and older adults with hip fractures.

### CONUT classification and corresponding mortality rates

To further clarify the relationship between nutritional status severity and short-term mortality, patients were stratified into four groups based on CONUT score: normal (0–1 points), mild malnutrition (2–4 points), moderate malnutrition (5–8 points), and severe malnutrition (9–12 points).

For 6-month mortality, a gradient trend was observed with increasing CONUT-based malnutrition severity. The severe malnutrition group had the highest 6-month mortality rate (33.33%), followed by the moderate malnutrition group (24.32%), the mild malnutrition group (11.90%), and the normal group (15.63%). Notably, the normal group showed a slightly higher mortality rate than the mild malnutrition group, but the overall trend indicated that worse nutritional status (higher CONUT classification) was associated with elevated short-term mortality risk.

For 12-month mortality, the gradient association between CONUT classification and mortality was more distinct. The severe malnutrition group still had the highest mortality rate (33.33%), while the moderate malnutrition group showed an increased mortality rate compared to the 6-month follow-up (32.43%). The mild malnutrition group’s 12-month mortality rate rose to 17.46%, and the normal group maintained a stable mortality rate of 15.63%. This result confirmed that moderate to severe malnutrition (CONUT score ≥5 points) was consistently linked to a significantly higher risk of 12-month mortality, while mild malnutrition and normal nutritional status were associated with relatively lower and more stable mortality rates.

Overall, the stratified analysis by CONUT classification revealed that malnutrition severity (assessed by CONUT score) was closely correlated with short-term postoperative mortality in middle-aged and older adults with hip fractures. Moderate to severe malnutrition identified by CONUT was a marker of high short-term mortality risk, providing clinical relevance for preoperative risk stratification.

### Patient characteristics and demographics by low CONUT (<3) and high CONUT (≥3)

The optimal cut-off value of the CONUT score for predicting short-term mortality was 2.5 according to the Youden index. Given that the CONUT score is calculated as an integer, a practical clinical cut-off value of ≥3 was used to stratify patients into low CONUT (<3, *n* = 69) and high CONUT (≥3, *n* = 129) groups for further analysis. As shown in Table 7, there were significant differences in multiple clinical and laboratory indicators between the two groups (all *p* < 0.05), while no statistically significant differences were observed in age, gender, surgical duration, hospitalization length, or most comorbidities (all *p* > 0.05).

**Table 7:** Patient characteristics and demographics by low CONUT (<3) and high CONUT (≥3).

Parameter	Low CONUT (*N* = 69)	High CONUT (*N* = 129)	*t*/*χ*^2^ value	*p*-value
Age	79.29 ± 11.08	76.98 ± 12.62	1.281	0.202
Male/female	29/40	42/87	1.753	0.186
Duration of surgery, median (Q1–Q3)	85 (63–104)	72 (55–97)	1.270	0.206
Hospitalization length, median (Q1–Q3)	11 (6–12)	10 (5–12)	0.150	0.881
Comorbidities	64 (92.75%)	104 (80.62%)	5.148	0.023
DM2	12 (17.40%)	21 (16.28%)	0.040	0.841
Hypertension	35 (50.72%)	62 (48.06%)	0.128	0.721
Pneumonia	13 (18.84%)	36 (27.91%)	1.984	0.159
SBP (mmHg)	155.36 ± 24.53	149.92 ± 26.28	1.418	0.158
DBP (mmHg)	79.17 ± 10.28	77.30 ± 12.50	1.063	0.289
WBC (10^9^/L)	10.01 ± 2.68	9.30 ± 2.51	1.842	0.067
Lymphocyte count (10^9/L)	1.07 ± 0.40	1.13 ± 0.48	0.833	0.406
Neutrophil count (10^9/L)	8.61 ± 2.26	7.56 ± 2.37	1.771	0.078
Hemoglobin (g/L)	123.00 ± 18.31	112.15 ± 23.39	3.344	0.001
Albumin (g/L)	39.62 ± 4.51	36.51 ± 5.56	3.990	0.000
ALP (u/L)	102.48 ± 93.15	85.88 ± 31.42	1.436	0.155
ALT (g/L)	20.49 ± 19.06	18.64 ± 13.00	0.802	0.423
Uric acid (μmol/L)	295.07 ± 100.49	322.41 ± 115.30	1.657	0.099
Blood calcium (mmol/L)	2.32 ± 0.14	2.21 ± 0.16	4.570	0.000
Serum phosphorus (mmol/L)	0.99 ± 0.19	1.01 ± 0.20	0.714	0.476
TC (mmol/L)	5.55 ± 0.87	3.78 ± 0.58	15.211	0.000
TG (mmol/L)	1.10 ± 0.52	0.99 ± 0.45	1.599	0.111
HDL (mmol/L)	1.54 ± 0.45	1.22 ± 0.31	5.259	0.000
LDL (mmol/L)	3.51 ± 0.96	2.30 ± 0.54	9.645	0.000

Data are presented as mean ± SD or as number (%). RDW, red blood cell distribution width. DM2, type 2 diabetic mellitus. SBP, systolic blood pressure. DBP, diastolic blood pressure. WBC, white blood cell. ALP, alkaline phosphatase. ALT, alanine aminotransferase. TC, total cholesterol. TG, triglyceride. HDL, high-density lipoprotein. LDL, low-density lipoprotein.

In terms of comorbidities, the proportion of patients with at least one comorbidity was significantly higher in the high CONUT group than in the low CONUT group (92.75% vs. 80.62%, *p* = 0.023), though the prevalence of specific comorbidities (DM2, hypertension, pneumonia) did not differ significantly (all *p* > 0.05). Regarding laboratory indicators, the high CONUT group had significantly lower hemoglobin (112.15 ± 23.39 g/L vs. 123.00 ± 18.31 g/L, *p* = 0.001), serum albumin (36.51 ± 5.56 g/L vs. 39.62 ± 4.51 g/L, *p* = 0.000), and blood calcium (2.21 ± 0.16 mmol/L vs. 2.32 ± 0.14 mmol/L, *p* = 0.000) compared to the low CONUT group.

In contrast, the high CONUT group had significantly higher uric acid levels (322.41 ± 115.30 μmol/L vs. 295.07 ± 100.49 μmol/L, *p* = 0.099, marginally significant) and markedly abnormal lipid profiles: total cholesterol (3.78 ± 0.58 mmol/L vs. 5.55 ± 0.87 mmol/L, p = 0.000), HDL (1.22 ± 0.31 mmol/L vs. 1.54 ± 0.45 mmol/L, *p* = 0.000), and LDL (2.30 ± 0.54 mmol/L vs. 3.51 ± 0.96 mmol/L, *p* = 0.000) were all significantly lower in the high CONUT group. Additionally, the high CONUT group tended to have lower white blood cell count (9.30 ± 2.51 × 10^9^/L vs. 10.01 ± 2.68 × 10^9^/L, *p* = 0.067) and neutrophil count (7.56 ± 2.37 × 10^9^/L vs. 8.61 ± 2.26 × 10^9^/L, *p* = 0.078), though these differences did not reach statistical significance.

No significant differences were observed between the two groups in terms of lymphocyte count, ALP, ALT, serum phosphorus, or triglyceride levels (all *p* > 0.05). These results indicate that patients with high CONUT scores (≥3 points) are characterized by poorer nutritional status (lower albumin, hemoglobin), abnormal calcium metabolism, and dyslipidemia, which may collectively contribute to the elevated short-term mortality risk observed in this subgroup.

### Mortality rates in the low-CONUT and high-CONUT groups

To quantify the impact of nutritional status (dichotomized by CONUT score) on short-term postoperative mortality, the 6-month and 12-month mortality rates were compared between the low CONUT group (<3 points) and high CONUT group (≥3 points).

At 6 months postoperatively, the mortality rate in the high CONUT group (20.16%) was significantly higher than that in the low CONUT group (5.80%). This indicates that patients with poor nutritional status (CONUT ≥3 points) had a more than threefold higher risk of death within 6 months compared to those with better nutritional status (CONUT <3 points).

Consistent with the 6-month results, the 12-month mortality rate remained significantly higher in the high CONUT group (25.58%) than in the low CONUT group (10.14%). Notably, the mortality gap between the two groups persisted and slightly widened over the extended follow-up period, confirming that the adverse impact of poor nutritional status on survival is sustained in the short term.

These data further validate the clinical significance of the CONUT score as a prognostic marker: stratifying patients by a CONUT cut-off value of 3 points effectively distinguishes between subgroups with significantly different short-term mortality risks. The high CONUT group consistently exhibited elevated mortality rates at both 6 and 12 months, highlighting the importance of preoperative nutritional assessment and targeted nutritional intervention for middle-aged and older adults with hip fractures.

## Discussion

Hip fracture is a leading cause of disability and short-term mortality in middle-aged and older adults, and preoperative risk stratification is critical for improving postoperative outcomes (12, 13). Nutritional and inflammatory status are considered modifiable prognostic factors in this population (14). Inflammation plays a critical role in promoting cellular injury and tissue damage. Emerging evidence has demonstrated that inflammatory signaling can trigger multiple forms of cell death and impair cellular function (15). In this retrospective cohort study, we compared the predictive value of CONUT, NLR, and PNI for 6-month and 12-month mortality in patients undergoing hip fracture surgery. The main findings were that the CONUT score was significantly associated with short-term mortality and showed better predictive performance than NLR and PNI, while NLR presented an inverse association and PNI showed no significant predictive value.

The CONUT score, which integrates serum albumin, total lymphocyte count, and total cholesterol, comprehensively reflects nutritional reserve, immune function, and metabolic status (10). Consistent with previous studies, our results indicated that a high CONUT score (poor nutritional status) was related to increased 6-month and 12-month mortality. The statistically optimal cut-off value for CONUT was 2.5, but we used ≥3 as the clinical threshold because CONUT is inherently an integer score. Rounding to 3 points improves clinical feasibility and ease of use in routine practice without significantly compromising prognostic performance. This approach is consistent with common clinical practice for nutritional risk screening tools. The optimal cutoff value of 2.5 (simplified to 3 points in clinical practice) effectively identified patients at higher risk. These findings support the use of CONUT in preoperative nutritional risk screening for hip fracture patients.

Recent studies have highlighted the prognostic value of nutritional indices in geriatric orthopedic trauma populations reported that PNI was associated with postoperative mortality in geriatric hip fracture patients (16). However, in the present study, PNI did not show significant predictive ability for short-term mortality, which may be due to differences in patient characteristics, follow-up duration, and surgical settings. These comparisons suggest that the predictive performance of nutritional scores may vary across populations.

In the present study, NLR did not show a satisfactory predictive value for mortality, which is inconsistent with the findings of most previous studies. This discrepancy may be explained by several factors. First, elderly patients with multiple comorbidities may present blunted systemic inflammatory responses after hip fracture. Second, the timing of blood sampling, baseline immune status, and hidden infections may influence NLR levels. Third, malnutrition-induced immune depression may reduce neutrophil elevation in response to surgical stress. This finding suggests that NLR should be interpreted cautiously in middle-aged and older patients with hip fracture.

In this retrospective cohort study, patients in the high CONUT group (≥3 points) exhibited distinct clinical characteristics, including lower hemoglobin, albumin, and blood calcium levels, as well as dyslipidemia (lower total cholesterol, HDL, and LDL). These abnormalities may collectively contribute to poor outcomes by impairing tissue repair, weakening immune defense, and disrupting metabolic balance (4). Notably, the high CONUT group also had a higher proportion of comorbidities, which aligns with the notion that malnutrition often coexists with multiple chronic conditions and exacerbates postoperative vulnerability (8). This underscores the need for integrated management of nutritional status and comorbidities in clinical practice.

In contrast, NLR showed limited predictive value in this study. Although NLR is widely recognized as a marker of systemic inflammation and immune imbalance (6), our results revealed an inverse association with mortality—non-survivors had lower NLR than survivors—which contradicts most previous reports. This discrepancy may be attributed to the specific characteristics of our study population, such as the high prevalence of comorbidities and variations in inflammatory responses to trauma and surgery. Additionally, the lack of significant predictive power for 6-month mortality suggests that NLR may not be sensitive enough to capture early postoperative risk, limiting its utility for short-term outcome prediction.

PNI, which has been validated as a prognostic indicator in various surgical populations (7), failed to show significant predictive value in our study. This finding differs from studies reporting PNI as an independent predictor of long-term mortality in hip fracture patients (9). The inconsistency may stem from differences in follow-up duration—our focus on short-term mortality (≤12 months) versus long-term outcomes in previous research. Additionally, the relatively narrow range of PNI values in our cohort (42.90–43.20) may have reduced its discriminatory ability. It is also possible that PNI, as a single composite index of albumin and lymphocyte count, is less comprehensive than CONUT in capturing the complex nutritional and metabolic abnormalities in middle-aged and older adults with hip fractures.

These findings may have potential clinical implications for the management of middle-aged and older adults with hip fractures. Preoperative assessment of CONUT score can help identify high-risk patients who may benefit from targeted nutritional interventions (17). For patients with CONUT ≥2.5 points, early nutritional support—such as increasing protein intake, supplementing essential nutrients, and correcting metabolic abnormalities—may improve postoperative outcomes (18). Additionally, the distinct laboratory characteristics of high CONUT patients (e.g., hypoalbuminemia, dyslipidemia) suggest potential therapeutic targets beyond general nutritional support, such as correcting calcium metabolism and lipid disorders. Besides, patients’ nutritional and inflammatory indicators are also important parameters that require attention (19).

Our study has several limitations. First, this was a single-center retrospective study, which may limit generalizability. Second, the influence of postoperative nutritional support was not recorded. Third, the sample size of patients with severe malnutrition was relatively small. Future multi-center prospective studies are needed to verify these findings.

In conclusion, the CONUT score appears to be a useful predictor for 6-month and 12-month mortality in middle-aged and older adults with hip fractures. Preoperative assessment of CONUT may help identify high-risk patients and guide individualized nutritional interventions. NLR and PNI show limited predictive value for short-term mortality in this population.

## Data Availability

The raw data supporting the conclusions of this article will be made available by the authors, without undue reservation.
